# The EpsE Flagellar Clutch Is Bifunctional and Synergizes with EPS Biosynthesis to Promote *Bacillus subtilis* Biofilm Formation

**DOI:** 10.1371/journal.pgen.1001243

**Published:** 2010-12-09

**Authors:** Sarah B. Guttenplan, Kris M. Blair, Daniel B. Kearns

**Affiliations:** Department of Biology, Indiana University, Bloomington, Indiana, United States of America; Agency for Science, Technology and Research, Singapore

## Abstract

Many bacteria inhibit motility concomitant with the synthesis of an extracellular polysaccharide matrix and the formation of biofilm aggregates. In *Bacillus subtilis* biofilms, motility is inhibited by EpsE, which acts as a clutch on the flagella rotor to inhibit motility, and which is encoded within the 15 gene *eps* operon required for EPS production. EpsE shows sequence similarity to the glycosyltransferase family of enzymes, and we demonstrate that the conserved active site motif is required for EPS biosynthesis. We also screen for residues specifically required for either clutch or enzymatic activity and demonstrate that the two functions are genetically separable. Finally, we show that, whereas EPS synthesis activity is dominant for biofilm formation, both functions of EpsE synergize to stabilize cell aggregates and relieve selective pressure to abolish motility by genetic mutation. Thus, the transition from motility to biofilm formation may be governed by a single bifunctional enzyme.

## Author Summary

Bacteria form persistent and antibiotic-resistant cell aggregates known as biofilms. Biofilms can form in environmental settings on plant and animal tissues, in industrial settings on pipes and the hulls of ships, and in clinical settings on catheters and medical devices. Biofilms are characterized by two features: the cells within the aggregates are non-motile, and they produce an extracellular polysaccharide (EPS) matrix. We have found a bifunctional enzyme EpsE that contributes to both features of biofilm formation in *Bacillus subtilis*. EpsE interacts with the flagella rotor to inhibit motility and also cooperates with other enzymes to synthesize the EPS matrix. Thus, the transition from motility to biofilm formation may be governed by a single bifunctional protein. In the past decade, research on biofilms has been focused on biofilm eradication. Understanding how cells transition into the biofilm state may provide additional approaches of preventing the formation of a biofilm in the first place.

## Introduction

In the environment, bacteria alternatively exist as planktonic individual cells or in cell aggregates known as biofilms [1], [2]. Planktonic cells can be either non-motile or motile by assembly and rotation of flagella. Cell motility often promotes initial biofilm formation but ultimately motility is inhibited during the transition to sessile cell aggregates [3]–[8]. Mature biofilms contain non-motile cells that are encapsulated in an extracellular matrix composed of polysaccharides, proteins, and DNA [9]. The mechanism of motility inhibition during biofilm formation is poorly understood and the underlying purpose for motility inhibition in biofilms is unknown.

The Gram positive soil bacterium *Bacillus subtilis* is a model organism for biofilm formation. *B. subtilis* biofilms manifest either as floating pellicles or as colonies with complex architecture. Both types of biofilms are stabilized by an extracellular polysaccharide matrix (EPS) and the amyloid protein TasA [10]–[12]. Production of both matrix components is tightly repressed by the DNA binding transcription factor SinR and a complex series of upstream regulators [13]–[16]. Notably, the 15 gene *eps* operon is directly repressed by SinR and encodes putative glycosyltransferases, presumably for EPS biosynthesis, as well as EpsE, a protein that inhibits flagellar rotation [17].

Flagella structure and function is best understood in the Gram negative bacteria *Escherichia coli* and *Salmonella enterica* [18], [19]. The flagellar basal body is anchored in the cell membrane and serves as a scaffold for the hook and the external, helical filament. Flagellar rotation occurs when an influx of protons through the MotA/MotB proton channel induces a conformational change in the motor protein MotA [20]–[22]. The conformation change in MotA is transduced into flagellar rotation by interaction with the ring of FliG rotor proteins that sits underneath the flagellar basal body [23]–[25]. EpsE is thought to directly interact with FliG to distort the MotA-FliG interaction and cut power to the flagellum like a molecular clutch [17]. The specific region of the EpsE protein that interacts with FliG is unknown.

EpsE also shows sequence similarity to the Type 2 family of glycosyltransferases and encodes the highly conserved DXDD active site motif [26], [27]. Here we demonstrate that EpsE has two genetically separable functions, acting both as an enzyme for EPS biosynthesis and acting as a clutch by protein-protein interaction. We further show that both functions of EpsE synergize to promote biofilm formation. Thus, an “ordinary” looking enzyme common to *eps* operons in diverse bacteria has an extraordinary additional function. Furthermore, the transition from motility to biofilm formation may be governed by a single protein.

## Results

### EpsE is a bifunctional glycosyltransferase

**Figure 1:**
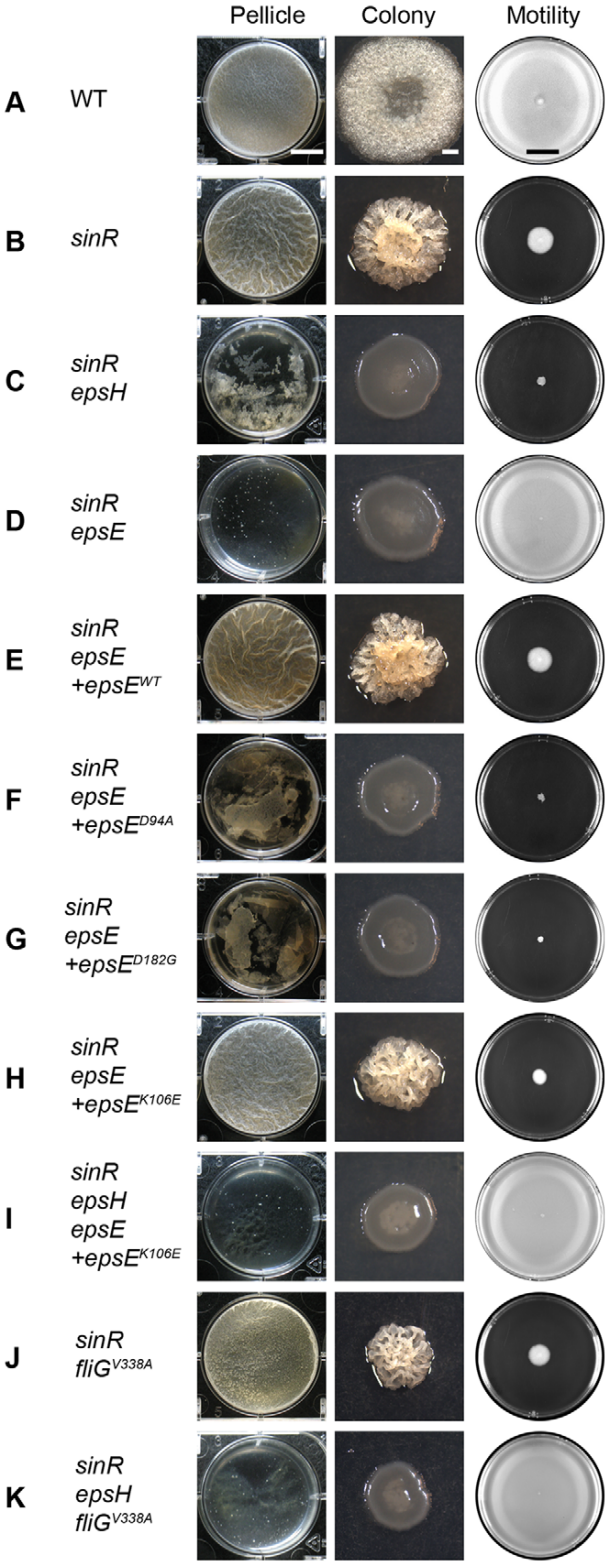
EpsE inhibits motility and promotes biofilm formation. The “pellicle” column depicts top-down images of 6-well microtiter plates containing MSgg media and the indicated strains incubated for 2 days at 25°C. Scale bar equals 1 cm. The “colony” column depicts colonies grown on MSgg agar for 3 days at 25°C. Scale bar equals 1 mm. The “motility” column depicts 0.7% LB agar plates centrally inoculated and incubated overnight at 37°C. Images were taken on a black background so zones colonized agar appear white, while zones of uncolonized agar appear black. Scale bar equals 2 cm. The indicated wild type and mutant strains are as follows: A) WT 3610, B) *sinR* DS859, C) *sinR epsH* DS1674, D) *sinR epsE* DS2174, E) *sinR epsE +epsE^WT^* DS2239, F) *sinR epsE +epsE^D94A^* DS2287, G) *sinR epsE +epsE^D182G^* DS5837, H) *sinR epsE +epsE^K106E^* DS4777, I) *sinR epsH epsE +^epsEK106E^* DS5142, J) *sinR fliG^V338A^* DS3005, and K) *sinR epsH fliG^V338^* ^A^ DS4532.

In *B. subtilis*, SinR represses the *eps* and *yqxM* operons, which are responsible for synthesizing the extracellular polysaccharide (EPS) and protein components of the extracellular matrix, respectively [10]–[12]. Consequently, a *sinR* mutant forms a colony with a more complex architecture and a thicker, more robust pellicle compared to wild type (Figure 1A and 1B). Mutation of either the EpsE or EpsH putative glycosyltransferases encoded within the *eps* operon disrupted complex colony architecture in the *sinR* background (Figure 1C and 1D). In pellicle assays a *sinR epsH* double mutant formed shattered sunken aggregates, but a *sinR epsE* double mutant completely abolished biofilm formation and aggregates did not accumulate (Figure 1C and 1D). We conclude that both glycosyltransferase homologs are required for biofilm formation, but that the absence of EpsE results in a more severe biofilm defect than the absence of EpsH.

To confirm that the severe biofilm defect in the *sinR epsE* double mutant was a direct consequence of the loss of *epsE*, the *epsE* gene was complemented at an ectopic site in the chromosome. To generate the complementation construct, the *epsE* gene was cloned downstream of the promoter of the *eps* operon (*P_eps_*) and inserted into the ectopic *amyE* site (*amyE::P_eps_-epsE*). Introduction of the P*_eps_*-*epsE* complementation construct rescued both complex colony architecture and pellicle formation to the *sinR epsE* double mutant (Figure 1E). EpsE encodes a highly conserved DXDD glycosyltransferase enzymatic active site motif (D^94^G^95^D^96^D^97^). To determine the contribution of the EpsE putative enzymatic active site to biofilm formation, aspartate^94^ (D94) was changed to an alanine residue (D94A) by site-directed mutagenesis of the *epsE* complementation construct (*amyE*::P*_eps_*-*epsE* ^D94A^). A *sinR epsE* mutant complemented with *epsE^D94A^* was severely reduced for both complex colony architecture and pellicle formation (Figure 1F). We conclude that the putative active site of EpsE is required for biofilms.

**Figure 2:**
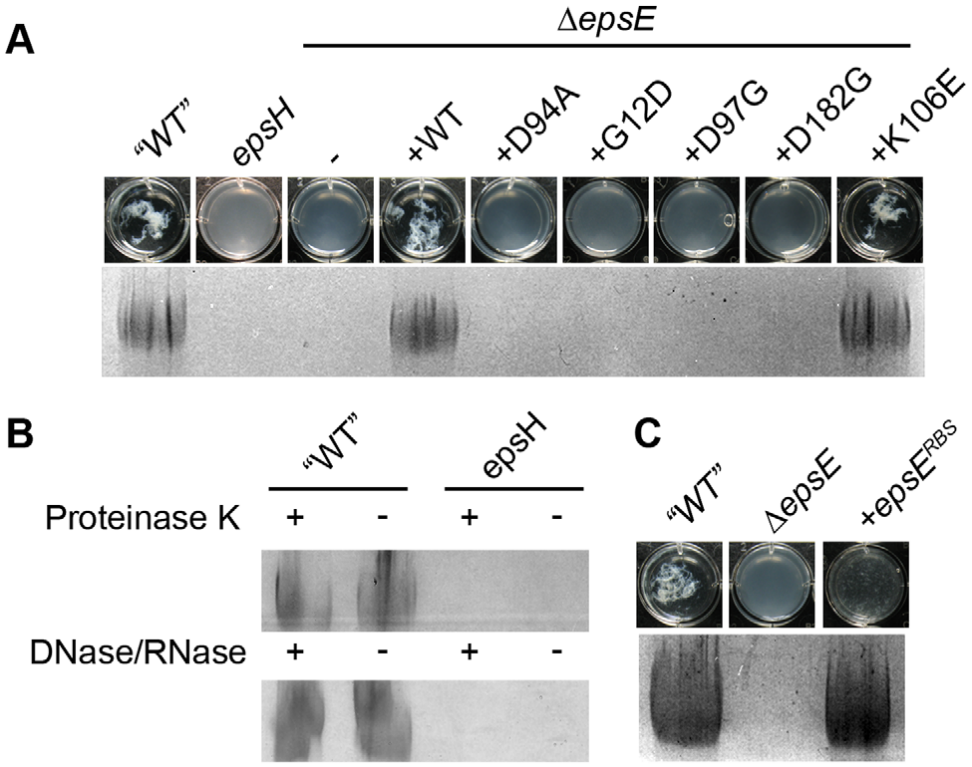
EpsE is required to produce EPS. A. Top row contains images of the chambers of a 12-well microtiter dish containing ethanol-precipitated supernatant from the indicated strain. Bottom row contains ethanol precipitated supernatant from the indicated strains resolved in the stacker of an SDS-PAGE gel stained with Stains-All. All strains contain *sinR::kan* and *tasA::Tn10 spec* mutations to increase expression of the *eps* operon and to liberate the EPS from the cell surface, respectively. The indicated wild type and mutant strains are as follows: “WT” (DS991), *epsH* (DS5187), *ΔepsE* (DS2369), *ΔepsE +epsE^WT^* (DS2370), *ΔepsE +epsE^D94A^* (DS2372), *ΔepsE +epsE^G12D^* (DS6191), *ΔepsE +epsE^D97G^* (DS6195), *ΔepsE +epsE^D182G^* (DS6190), and *ΔepsE +epsE^K106E^* (DS6312). B. The indicated “WT” (DS991) and *epsH* (DS5187) supernatants were treated with (+) or without (−) Proteinase K or DNase and RNase, ethanol precipitated, and resolved in the stacker of an SDS-PAGE gel stained with Stains-All. C. Top, wells contain precipitated supernatant. Bottom, stacking gel contains precipitate resolved by SDS-PAGE and stained with Stains-All. The indicated wild type and mutant strains are as follows: “WT” (DS991), *ΔepsE* (DS2369), and *ΔepsE +epsE^RBS^* (DS7147).

One way in which the EpsE putative enzymatic activity could contribute to biofilm formation is by the synthesis of the EPS matrix component. To determine whether EPS was being synthesized, EPS was first isolated and purified from cells wild type for EpsE. To improve EPS recovery, EPS synthesis was enhanced by mutation of SinR, and EPS was liberated from the cell surface by mutation of the EPS extracellular organizing protein TasA. When spent media was harvested from dense cultures of a *sinR tasA* double mutant and mixed with ethanol, a threadlike substance precipitated (Figure 2A). When the precipitate was resolved by SDS-polyacrylamide gel electrophoresis (PAGE) and stained with Stains-All, a band that did not leave the stacking gel was present, consistent with a high molecular weight substance (Figure 2A) [28]. This precipitate was insensitive to treatment with Proteinase K, DNase, and RNase, indicating that the substance was not composed of protein, DNA, or RNA (Figure 2B).

**Figure 3:**
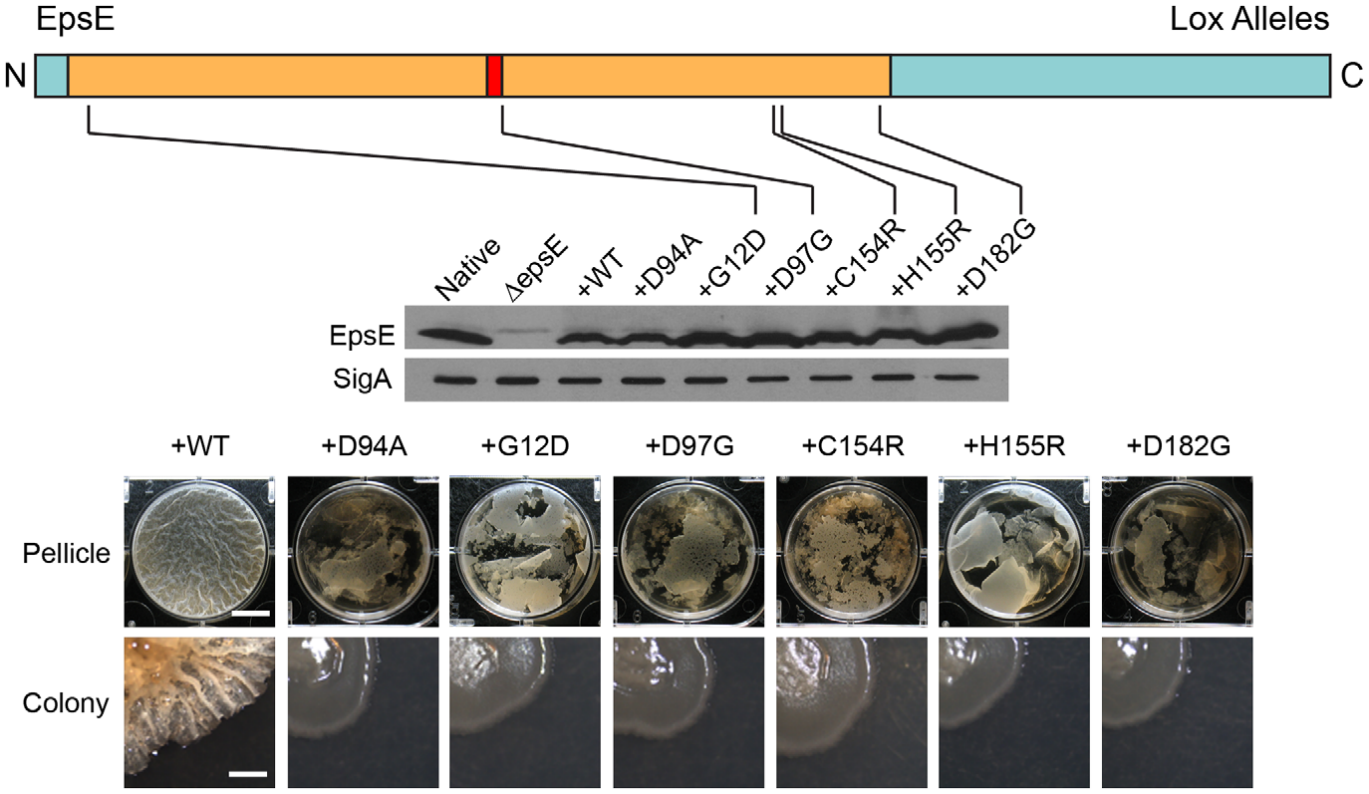
Point mutations in EpsE that abolish biofilm formation. EpsE primary structure is depicted as a cartoon with the amino terminus (N) and carboxy terminus (C) labeled and the loss-of-extracellular polysaccharide (*lox*) mutations at the indicated positions. The tan region corresponds to the region of EpsE with homology to glycosyltransferases, with the red region corresponding to the conserved active site. Blue indicates the regions of EpsE not conserved with other proteins. Whole cell lysates of cells mutated for *sinR epsH* and the indicated *epsE* alleles were separately probed with anti-EpsE antibody and anti-SigA antibody (to serve as a loading control). The indicated strains are as follows for the western blot: Native (DS1674), ΔepsE (DS2946), +WT (DS3844), +D94A (DS4593), and +*lox* mutants DS5848, DS5841, DS5839, DS5852, and DS5837. The “pellicle” row depicts top-down images of 6-well microtiter plates containing MSgg media and the indicated strains were incubated for 2 days at 25°C. Scale bar equals 1 cm. The “colony” row depicts colonies grown on MSgg agar incubated for 3 days at 25°C. Scale bar equals 1 mm. The indicated wild type and mutant strains are as follows: +*epsE^WT^* (DS2239), *+epsE^D94A^* (DS2287), *+epsE^G12D^* (DS5848), *+epsE^D97G^* (DS5841), *+epsE^C154R^* (DS5839), *+epsE^H155R^* (DS5852), and *+epsE^D182G^* (DS5837).

Mutation of either EpsE or EpsH abolished the production of the threadlike precipitate and abolished the high molecular weight Stains-All-reactive material (Figure 2A). The precipitate and Stains-All-reactive material was restored to the *sinR tasA epsE* triple mutant when complemented with wild type *epsE*, but not when complemented with the EpsE glycosyltransferase active site mutant allele *epsE^D94A^* (Figure 2A). The active site allele mutant protein was produced in amounts similar to wild type and thus the loss of precipitate was not due to a loss of EpsE protein (Figure 3). We infer that the precipitate and high molecular weight Stains-all-reactive material is the EPS that constitutes the biofilm matrix and that EpsE is required for its synthesis. Furthermore, the putative EpsE active site is essential for EPS synthesis and we infer that EpsE acts as a glycosyltransferase enzyme to promote biofilm formation.

Here we show that EpsE likely acts as an enzyme to synthesize EPS, but EpsE has also been reported to interact with the flagellar rotor component FliG and act as a clutch to inhibit flagellar rotation [17]. The *epsE^D94A^* active site mutant allele restored motility inhibition to a *sinR epsE* double mutant, even though it lacked EPS biosynthesis (Figure 1D and 1F). We conclude that the EpsE putative enzymatic activity is unrelated to clutch function and we hypothesize that EpsE is bifunctional. To further demonstrate genetic separability of the two functions we randomly mutagenized EpsE and screened for loss-of-extracellular polysaccharide (*lox*) alleles that only abolished EPS production, and loss-of-clutch (*loc*) alleles that only abolished motility inhibition.

### Residues required for EpsE enzymatic activity

To support genetic screens involving EpsE, a random pool of mutations (*epsE^mut^*) was generated by amplifying the *epsE* gene by low fidelity polymerase chain reaction (PCR), cloned downstream of the native *P_eps_* promoter, and inserted into a plasmid between the arms of the *amyE* gene (*amyE::P_eps_-epsE^mut^*). The plasmid library was then integrated into the *B. subtilis amyE* locus as a pool of mutants. Thus, the mutated copies of *epsE* were incorporated in a manner analogous to the *epsE* complementation construct, and *epsE* mutant alleles could be screened for either the ability or inability to complement an *epsE* deletion.

The screen for loss of extracellular polysaccharide (*lox*) mutant alleles of *epsE* was conducted in two sequential stages. The first stage of the screen isolated *epsE* mutants defective in EPS production. The *epsE^mut^* mutant pool was introduced into a *sinR epsE* double mutant and colony morphology was analyzed. Colonies containing *epsE* alleles that had the ability to produce EPS had a rough colony phenotype and were excluded from further screening [29], [10]. Colonies containing *epsE* mutants that did not have the ability to produce EPS had a smooth colony phenotype and were chosen for the second stage of the screen. Each smooth *epsE* colony was individually inoculated onto swarm agar plates to determine if the allele of EpsE was proficient for clutch function and motility inhibition. Motile strains, which lost clutch function, were excluded from further study to avoid EpsE null mutants. Thirty mutants that were inhibited for motility and had a smooth colony phenotype were isolated as *lox* alleles.

Sequencing of the *lox* mutants revealed three classes of mutations within the *epsE* gene after discarding siblings (Table S1). Class 1 mutations were located within the conserved DXDD glycosyltransferase active site motif (Figure 3, Table S1). Class 2 mutations were located outside of the conserved DXDD motif but were presumably important in either substrate recognition or substrate specificity (Figure 3, Table S1). For example, one particular class 2 mutant changed the conserved asparate^182^ to a glycine (EpsE^D182G^), and the homologous aspartate was found to be important for nucleotide binding in ExoM of *Sinorhizobium meliloti* [27]. Another class 2 mutant altered histidine^155^ that was highly conserved among other glycosyltransferases [30]. The residues glycine^12^ and cysteine^154^ were not conserved in other glycosyltransferases, but the substitutions at these positions may either confer substrate specificity or interfere with the conserved residues adjacent to them (Figure S1A). Consistent with a defect in glycosyltransferase function, no EPS was isolated from supernatants of cells containing either *lox* class 1 or class 2 mutations (Figure 2A). Similar to the EpsE^D94A^ active site mutation, Class 1 and 2 mutants synthesized wild type amounts of EpsE protein (Figure 3). Class 3 mutations contained more than one substitution within the EpsE coding region. We note however, that each Class 3 mutation contained a substitution corresponding to a position already identified in either Class 1 or Class 2 alleles. We infer that only one of the substitutions in Class 3 mutants actually accounts for the loss of EPS phenotype. Mutants in all *lox* classes abolished EPS biosynthesis but retained clutch activity.

### Residues required for EpsE clutch activity

The EpsE loss-of-clutch (*loc*) screen was conducted in three sequential stages to isolate alleles of *epsE* that were defective in motility inhibition, retained EPS biosynthetic capacity, and produced EpsE protein levels comparable to wild type. In the first stage, *epsE* mutants were isolated that were defective in motility inhibition. The pool of *epsE^mut^* alleles were introduced to a *sinR* mutant deleted for the entire *eps* operon, pooled, and inoculated in the center of a swarm agar plate. Deletion of the entire *eps* operon ensured that EPS biosynthesis could not be restored regardless of the *epsE* allele introduced. Alleles of *epsE* that were functional for the clutch inhibited motility, remained at the site of inoculation, and were excluded from further screening. In contrast, alleles of *epsE* that were defective for the clutch remained motile, spread from the site of inoculation, and were harvested.

In the second stage, *epsE* alleles from the first stage were screened for those that retained EPS biosynthetic capacity by introduction to a *sinR epsE* double mutant and plating for discrete colony forming units. Alleles of *epsE* non-functional for EPS biosynthesis exhibited a smooth colony morphology and were excluded from further screening. In contrast, alleles of *epsE* that were functional for EPS biosynthesis restored rough colony architecture and were individually isolated. In this particular genetic background, motility was inhibited due to the dominance of EPS production and therefore the EPS proficient *epsE* alleles were backcrossed into a *sinR epsE epsH* triple mutant to ensure that motility inhibition was still abolished (representative *epsE* allele Figure 1H and 1I).

**Figure 4:**
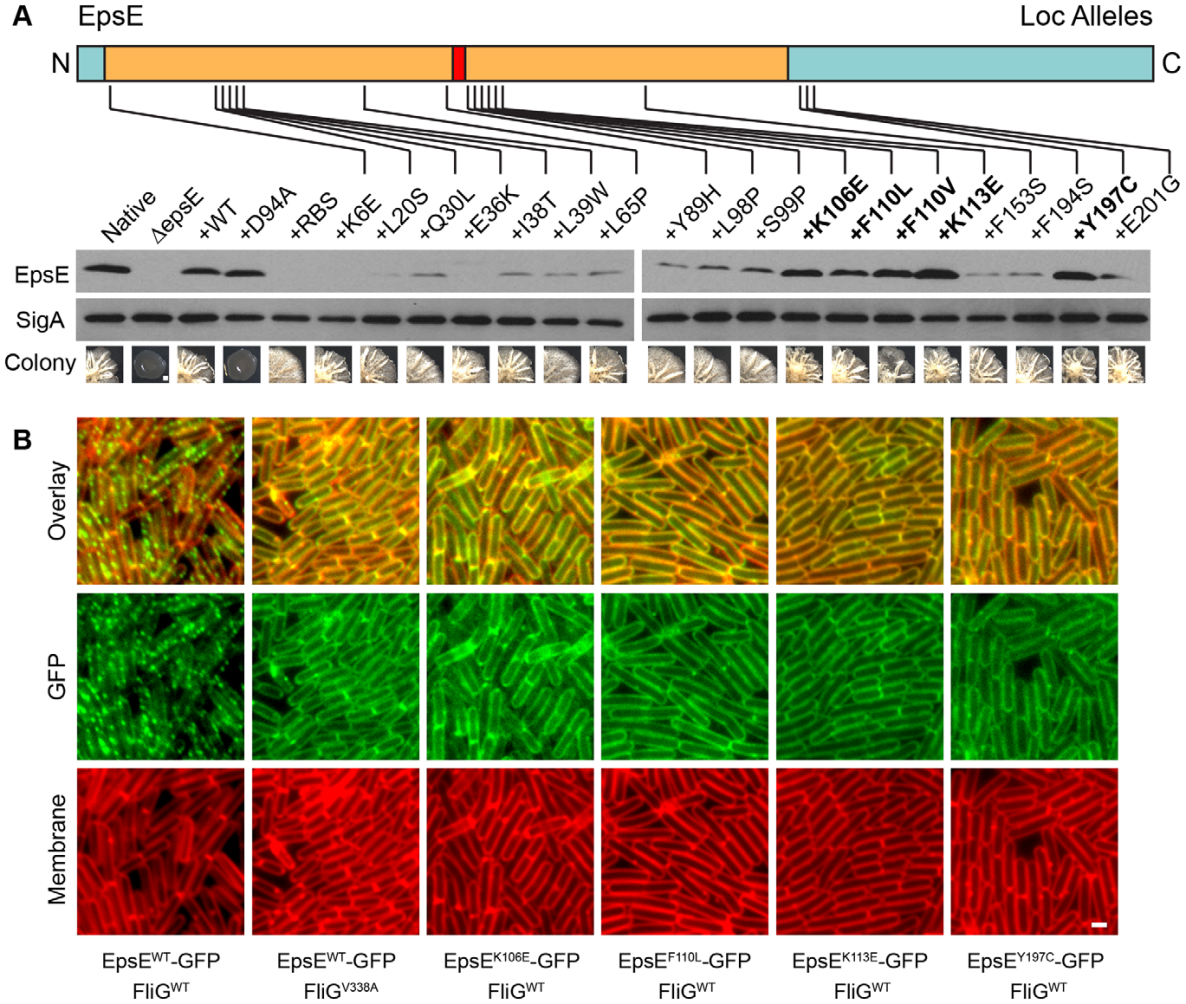
Point mutations in EpsE that abolish clutch function. A) EpsE primary structure is depicted as a cartoon with the amino terminus (N) and carboxyl terminus (C) labeled and the mutations generated during the loss-of-clutch (*loc*) screen at the indicated positions. The tan region corresponds to the region of EpsE with homology to glycosyltransferases, with the red region corresponding to the conserved active site. Blue indicates the regions that have little to no conservation to other proteins. The specific mutations that meet all three criteria for *loc* alleles (loss of motility inhibition, maintenance of EPS production, and wild type levels of EpsE protein) are bolded. Whole cell lysates of cells mutated for *sinR epsH* and the indicated *epsE* alleles were separately probed with anti-EpsE antibody and anti-SigA antibody (to serve as a loading control). The indicated strains are as follows for the western blot: Native (DS1674), ΔepsE (DS2946), +WT (DS3844), +D94A (DS4593), and +*loc* mutants (DS5086, DS5087, DS5088, DS5089, DS5090, DS5135, DS5136, DS5137, DS5138, DS5139, DS5140, DS5141, DS5142, DS5143, DS5144, DS5145, DS5153, DS5154, and DS5185). To generate the colony images, cells mutated for *sinR* (but wild type for *epsH*) and the indicated *epsE* alleles were inoculated onto MSgg agar plates and incubated for 3 days at 25°C. The scale bar equals 1mm. The indicated strains are as follows for the colony images: Native (DS859), ΔepsE (DS2174), +WT (DS2239), +D94A (DS2287), and +*loc* mutants (DS4275, DS4284, DS4289, DS4689, DS4697, DS4698, DS4729, DS4733, DS4734, DS4738, DS4741, DS4742, DS4743, DS4746, DS4747, DS4748, DS4777, DS4778, and DS4782). B. Fluorescent micrograph series in which GFP is fused to the indicated alleles of EpsE and false colored in green. Membranes are false colored in red. All strains contain a mutation in *sinR* and a deletion of the native copy of *epsE*. All *epsE* alleles were complemented at the *thrC* ectopic locus. FliG mutations, if any, are at the native site. The indicated strains are as follows: EpsE^WT^ FliG^WT^ (DS2989), EpsE^WT^ FliG^V338A^ (DS3004), EpsE^K106E^ FliG^WT^ (DS5156), EpsE^F110L^ FliG^WT^ (DS5489), EpsE^K113E^ FliG^WT^ (DS5491), and EpsE^Y197C^ FliG^WT^ (DS5155). Scale bar equals 4 µm.

Ultimately, after two stages of screening we had retained a set of sixty *epsE* alleles that were deficient for motility inhibition but proficient for EPS synthesis. Sequencing of these mutants revealed a variety of mutations in the *epsE* gene as well as in the ribosome binding site (RBS). Some alleles contained multiple mutations and were discarded from further analysis, while others contained the same mutation (siblings) and only one of each sibling was retained for further study. The remaining alleles were advanced to the third stage of the screen in which EpsE protein levels were assayed (Figure S2). To establish a “wild type” standard for EpsE protein comparison, whole cell lysates of cells mutated for *sinR* (to increase *eps* operon expression) and *epsH* (to prevent cell aggregation), were resolved by SDS-PAGE, electroblotted, and probed with an anti-EpsE antibody. A high level of EpsE was produced in the *sinR epsH* double mutant and this established the baseline for EpsE comparison (lane “Native”) (Figure 4A). Cells triply mutated for *sinR*, *epsH*, and *epsE* did not produce EpsE, but ectopic complementation restored EpsE levels comparable to wild type (*+WT*) (Figure 4A). We conclude that ectopic complementation restored native levels of EpsE.

To assay the amount of EpsE produced by the mutant alleles, we used the *sinR epsH epsE* triple mutant and complemented the strain with the various *epsE* mutants (*amyE::P_eps_-epsE^mut^*). Many of the screened clutch-defective mutant alleles, including mutations in the *epsE* RBS, resulted in a reduced amount of EpsE relative to the wild type standard (Figure 4A). Four alleles (K106E, F110L, F110V, and K113E) near the DXDD enzymatic active site sequence, and one allele in the C terminal region of EpsE (Y197C), however, produced EpsE levels comparable to the wild type (Figure 4A). Whereas many alleles abolished motility inhibition while retaining EPS synthesis, only these latter 5 alleles met the definition of the loss-of-clutch (*loc*) genotype as their phenotype was not due to a reduction in protein levels (Figure 2, Figure 4A). We hypothesize that the *loc* alleles represent residues likely required for interaction with FliG.

Clutch function is correlated with a punctate localization of EpsE-GFP to the cell membrane that can be abolished by mutations of FliG that renders FliG clutch-insusceptible (e.g. FliG^V338A^) [17] (Figure 4B, Figure S3). To determine the cellular localization pattern of the *loc* alleles, translational fusions were constructed between each allele and GFP, and placed at an ectopic locus (*thrC:: P_eps_-epsE^loc^-GFP*). In each case, the EpsE^loc^-GFP fusions displayed a diffuse membrane-associated pattern of localization reminiscent of the localization of EpsE-GFP in the FliG clutch-insusceptible background (Figure 4B). Thus, punctate localization of EpsE is tightly correlated with clutch activity and either *loc* mutations in EpsE or clutch-insusceptible mutations in FliG mislocalize EpsE and render the cells motile. We conclude that the residues mutated in the *loc* alleles are required for interaction with FliG.

### Two functions of EpsE synergize to promote pellicle formation

EPS biosynthesis has been shown to be required for biofilm formation and the inhibition of motility has been speculated to stabilize cell aggregates [29], [17]. To determine the relative contribution of the EPS synthesis and clutch activities of EpsE to biofilm formation, we constructed strains that were disrupted for one or the other function. All strains were mutated for *sinR* to alleviate repression on the *eps* operon, the native copy of *epsE* was deleted, and alleles of *epsE* were complemented at the *amyE* locus (*amyE::P_eps_-epsE*). Whereas the EpsE*^lox^* mutant (D94A) was specifically defective for EPS biosynthesis and formed a crippled pellicle that shattered and sank to the bottom of the tank, the EpsE*^loc^* mutant (K106E) formed a pellicle indistinguishable from a *sinR* mutant (Figure 1F and 1H). We conclude that the contribution of EpsE to EPS biosynthesis is more significant than the contribution of clutch function for pellicle formation.

We next attempted to determine the consequence of disrupting both EPS biosynthesis and clutch function simultaneously and in a manner that did not depend on the allele of EpsE. For example, a *sinR epsH* double mutant formed a shattered pellicle reminiscent of a *sinR* mutant containing an EpsE*^lox^* allele (Figure 1C and 1F or 1G). Also, a *sinR* mutant containing a *fliG^V338A^* clutch-insusceptible allele formed a robust pellicle reminiscent of a *sinR* mutant containing an EpsE*^loc^* allele (Figure 1J and 1H). When a *fliG^V338A^* mutation and an *epsH* mutation were simultaneously introduced into a *sinR* mutant, only small flecks of biomass accumulated in the well and the phenotype was more severe than either mutation alone (Figure 1K). A similar phenotype resulted when a *sinR* mutant was simultaneously mutated for *fliG^V338A^* and *epsE^D94A^* (Figure S4B). Thus, different methods of simultaneously disrupting EPS biosynthesis and clutch function produced a severe defect in pellicle formation reminiscent of a *sinR* mutant that lacks both functions due to a complete deletion of the *epsE* gene (Figure 1D). We conclude that both functions of EpsE synergize to promote pellicle formation.

**Figure 5:**
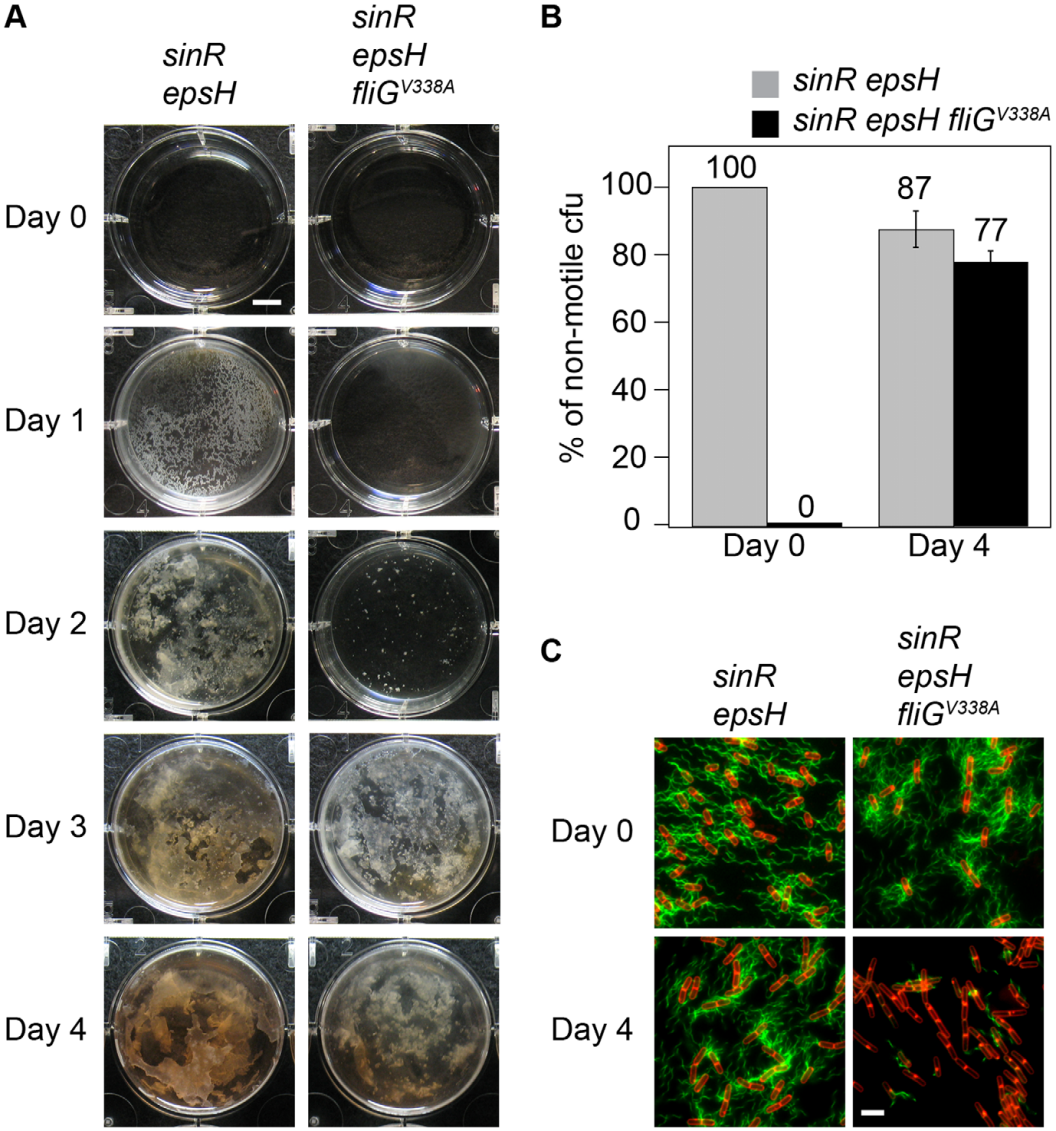
Motility inhibition enhances biofilm formation. A) The pellicle images are top views of DS1674 (*sinR epsH*) and DS4532 (*sinR epsH fliG^V338A^*) grown at 25°C for the indicated time period. B) The graph represents the percentage of isolated colonies from the indicated strains that were non-motile when dilution plated on Day 0 and Day 4. Error bars are the standard deviation of three replicates. C) Fluorescent micrographs of the parent strains and isolates from 4 days of growth stained with FM4-64 (membrane, false colored red) and Alexa Fluor C_5_ 488 maleimide (flagella, false color green). All strains express the modified flagellar filament protein Hag^T209C^. The following strains were used to generate this figure: *sinR epsH* (DS2179), *sinR epsH fliG^V338A^* (DS7289), *sinR epsH* 4 day isolate (DS7290), and *sinR epsH fliG^V338A^ saf* non-motile isolate (DS7293). Scale bar equals 2µm.

Upon prolonged incubation, the *sinR epsH fliG^V338A^* mutant developed *saf* suppressors (suppressors of aggregate formation) that restored biomass reminiscent of a *sinR epsH* mutant (Figure 5A). One way in which large aggregates could be restored was if the suppressor mutations had somehow abolished flagellar motility. To determine whether the suppressors were motile, we grew pellicles of a *sinR epsH* and a *sinR epsH fliG^V338A^* mutant, harvested the pellicles from both strains at day 4, dilution plated, and inoculated individual colonies onto swarm agar (Figure 5A). The *sinR epsH* population was initially non-motile, but we found that a small percentage of isolated colonies had gained motility upon prolonged incubation in the pellicle assay, likely through loss of EpsE or EpsE activators [17], [16] (Figure 5B). Whereas the *sinR epsH fliG^V338A^* triple mutant population was initially motile, a large percentage of isolated colonies had lost motility concomitant with aggregate formation (Figure 5B). We infer that there is strong selective pressure to form aggregates and that loss of motility is necessary for aggregate formation.

One way in which motility could be abolished in the absence of the flagellar clutch was by mutation of a gene required for flagellar assembly. To determine whether the *saf* suppressors of the *sinR epsH fliG^V338A^* triple mutant were defective for flagella, we introduced a modified version of the flagella filament that can be fluorescently labeled (*amyE::P_hag_-hag^T209C^*) [17]. Whereas the *sinR epsH fliG^V338A^* parent strain was motile, the *saf* mutants had a severely decreased number of flagella that were not sufficient for motility (Figure 5C). In comparison, the clutch proficient *sinR epsH* strain did not lose the ability to assemble flagella after prolonged incubation (Figure 5C). We infer that the non-motile *saf* suppressors of *sinR epsH fliG^V338A^* triple mutant have a mutation in an essential gene for flagellar synthesis and/or function, and that the severe decrease in the number of functional flagella restored aggregate formation. Thus, motility is detrimental to aggregate and pellicle formation, and the clutch relieves genetic selective pressure for motility loss that could otherwise result in mutation of any of the 40 genes required for flagella biosynthesis.

## Discussion

In many bacteria, the motility-to-biofilm transition is thought to be complex [2]. Here we demonstrate in *B. subtilis* that the motility-to-biofilm transition may be potentially reduced not only to the regulation of a single *eps* operon, but to a single protein within the operon, EpsE, that is bifunctional. EpsE is a glycosyltransferase that participates as an enzyme to synthesize the biofilm EPS. Furthermore, EpsE acts as a flagellar clutch to inhibit motility through interaction with the flagellar rotor. The two functions of EpsE are genetically separable, and through separate and distinct mechanisms, EpsE promotes matrix synthesis and inhibits motility to synergistically stabilize the biofilm.

The two functions of EpsE are mechanistically separable as indicated by the phenotype of mutations in the EpsE ribosome binding site (RBS) (Figure 4A). The RBS mutants reduced the level of EpsE protein below the limit of detection, but nonetheless retained the ability to produce EPS and form colonies with complex architecture (Figure 2C, Figure 4A). We infer the mechanism of EPS biosynthesis is independent of protein levels and therefore EpsE acts sub-stoichiometrically like an enzyme. The RBS mutants and other mutations that reduced EpsE protein levels, however, resulted in a loss of motility inhibition. We infer that the mechanism of clutch function is dependent on protein levels and therefore EpsE acts stoichiometrically, via a protein-protein interaction. EpsE is thought to interact directly with FliG, and for each basal body, approximately 26 FliG subunits polymerize into a wheel-like rotor [31]–[33]. The number of EpsE molecules that must interact with the rotor to inhibit rotation of a flagellum is finite but unknown.

**Figure 6:**
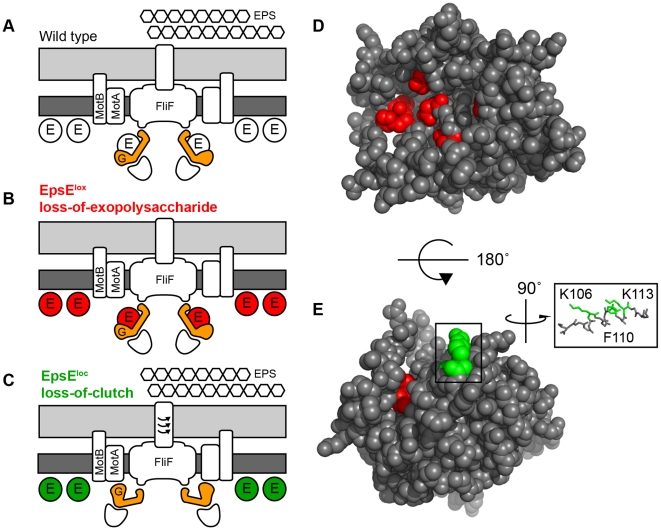
EpsE is a bifunctional protein that contributes to EPS biosynthesis and acts as a clutch on the flagella rotor. A–C. Cross-section diagrams of the *B. subtilis* flagellar basal body. Dark grey rectangles indicate the plasma membrane while light grey rectangles indicate the peptidoglycan cell wall. “G” indicates the rotor FliG. “E” indicates EpsE. Hexagons indicate the biofilm EPS matrix. Red and green circles indicate *lox* and *loc* alleles of EpsE respectively. A) Wild type cells are capable of EPS biosynthesis and EpsE disables flagellar rotation by disrupting the FliG-MotA interaction. B) Cells containing EpsE^Lox^ (Red) are incapable of EPS biosynthesis, but EpsE still acts as a clutch on FliG. C) Cells containing EpsE^Loc^ (Green) are capable of EPS biosynthesis, but are unable to interact with FliG and act as a clutch. D–E. Spacefilling model of predicted EpsE structure. Red indicates the location of *lox* mutations while green indicates the location of *loc* mutations. Inset focuses on the indicated alpha helix showing the three *loc* residues K106, F110, and K113 that localize to the same face.

The two mechanisms of EpsE may also be distinguished by subcellular localization patterns. EpsE localized both as membrane associated-puncta as well as a diffuse membrane-associated haze (Figure 6A–6C). Punctate localization is associated with clutch activity as clutch-insusceptible mutations in FliG or loss-of-clutch alleles in EpsE abolished puncta formation. While it is unknown whether EpsE in puncta participate in enzymatic reactions, punctate localization was not required for EPS biosynthesis. EPS biosynthesis occurs at the cytoplasmic membrane and we hypothesize that the diffuse membrane localization may represent interactions with other EPS enzymes or substrates [34].

The two mechanisms of EpsE may be further distinguished by mapping the location of the residues required for each activity in the tertiary structure. EpsE is an insoluble protein and has proven difficult to purify for structural analysis. Instead, we generated a predicted three dimensional structure of EpsE by threading the primary sequence through known glycosyltransferase structures, and the *lox* and *loc* residues were mapped onto the model (Figure 6D–6E). In partial validation of the predicted structure, the DXDD motif and *lox* residues were located in a pocket consistent with the active site found in other glycosyltransferases (Figure 6D) [26], [35]. Three of the four *loc* residues, however, localized to the same face of a predicted alpha helix within a groove on the exterior of the protein (Figure 6E). While the fourth *loc* residue was not contained within the homology model, we hypothesize that the remaining residues constitute the site of contact with FliG.

EpsE is part of a growing list of enzymes that regulate other functions in the cell by direct protein-protein interaction. For example, GlnA acts enzymatically as glutamine synthetase in *B. subtilis*, and also controls transcriptional regulators of nitrogen metabolism by direct protein-protein interaction [36], [37]. Glycosyltransferases are becoming increasingly identified as having additional regulatory functions. In *B. subtilis*, UgtP acts as a glucosyltransferase for glucolipid synthesis and also inhibits polymerization of the cell division protein FtsZ by protein interaction [38], [39]. In *Listeria monocytogenes*, GmaR enzymatically glycosylates the flagellum and directly interacts with the transcription factor MogR to derepress flagellar gene expression [40]–[42]. We note that many bacteria encode EPS operons that are rich in glycosyltransferase homologs, and that pleiotropic effects often observed in biofilm regulatory mutants may be due to as-yet-unidentified bifunctional enzymes. Bifunctional enzymes are important proteins that couple metabolism to related physiological functions. Here we show that EpsE couples EPS biosynthesis and functional control of the flagellum during biofilm formation.

Functional control of the flagellum has been implicated in the biofilm formation of bacteria besides *B. subtilis*. In *Escherichia coli*, the protein YcgR is a c-di-GMP dependent inhibitor of swimming speed and chemotaxis [43]–[45]. Like EpsE, YcgR may be involved in the biofilm transition because c-di-GMP is a ubiquitous transition regulator that inhibits motility and promotes biofilm formation [46], [7]. In *Pseudomonas aeruginosa*, SadC is a putative c-di-GMP synthase and positive regulator of biofilm formation by promoting EPS production. Mutation of SadC results in an increase in flagellar rotation reversal frequency that has been associated with surface attachment [47], [48]. Thus, inhibition of flagellar function may play an important role in the transition from motility to biofilm formation, as the pre-existent flagella are regulated faster than can be accomplished by transcriptional control.

Here we show that functional inhibition of the flagellum is critical for aggregate formation of cells crippled for EPS biosynthesis. The clutch may be most relevant in the natural environment, during early biofilm formation, or when biofilm formation is otherwise impaired. We demonstrate the synergy of the clutch and EPS enzymatic activity in a *sinR* mutant that produces a uniform population of cells that express flagellar genes [49]. In the wild type, however, mutation of the clutch function, either by deletion of *epsE* or introduction of a clutch insusceptible allele of FliG, does not show the severe synergistic effect on the biofilm (Figure S4A). Wild type planktonic populations are mixtures of cells that are on and off for flagellar gene expression and we infer that in the wild type, a population of non-motile cells is pre-existent and therefore does not need to make the motility-to-biofilm transition [50]. We further infer that the clutch is still relevant and important for the motile subpopulation to be included in the aggregates. In support of this assumption, we find that there is strong genetic selective pressure to abolish motility concomitant with aggregate formation (Figure 5). Thus, the clutch may not only inhibit motility to form the biofilm but may also relieve selective pressure on mutation of any of approximately 40 genes required for flagellar synthesis.

EpsE is the first clutch protein discovered for the bacterial flagellum and other flagellar clutches may be difficult to identify [17]. For example, the EpsE protein does not contain a conserved domain commonly associated with protein-protein interactions nor would one predict from the primary sequence that the EpsE protein was related to the flagellum. Rather, much of the protein sequence is conserved among other glycosyltransferases, including some of the residues we identify as required for clutch function (Figure S1A). Interestingly, three of the four residues that render FliG susceptible to the clutch are conserved in most other bacteria but not in *B. subtilis* [17] (Figure S1B). We infer that, at least in the case of *B. subtilis*, the FliG protein may have evolved to accommodate inhibition by a protein present during biofilm formation (i.e. EpsE). Perhaps the FliG in each bacterium evolved separately to modify different sites such that each clutch protein is uniquely suited to disable the rotor under specific conditions. Taken together, we conclude that it will be difficult to find functional inhibitors of the flagellum using bioformatic analyses alone and that clutch proteins may be best discovered by classical genetic approaches.

## Materials and Methods

### Strains and growth conditions

*B. subtilis* strains were grown in Luria-Bertani (LB) (10 g tryptone, 5 g yeast extract, 5 g NaCl per L) broth or on LB plates fortified with 1.5% Bacto agar at 37°C. When appropriate, antibiotics were included at the following concentrations: 10 µg/ml tetracycline, 100 µg/ml spectinomycin, 5 µg/ml chloramphenicol, 5 µg/ml kanamycin, and 1 µg/ml erythromycin plus 25 µg/ml lincomycin (*mls*).

For pellicle formation experiments, 10 µl of culture grown overnight at room temperature in LB medium was inoculated into 10 ml minimal MSgg medium (5 mM potassium phosphate (pH 7), 100 mM MOPS (pH 7), 2 mM MgCl_2_, 700 µM CaCl_2_, 50 µM MnCl_2_, 50 µM FeCl_3_, 1 µM ZnCl_2_, 2 µM thiamine, 0.5% glycerol, 0.5% glutamate, 50 µg/ml tryptophan, 50 µg/ml phenylalanine, and 50 µg/ml threonine) in 6-well microtiter plates and incubated at 25°C for 2 days [29]. For colony architecture analysis, colonies were toothpick inoculated onto minimal MSgg medium fortified with 1.5% Bacto agar and incubated for 3 days at 25°C.

For the motility assay, swarm agar plates containing 25 ml LB fortified with 0.7% Bacto agar were prepared fresh and the following day were dried for 20 minutes in a laminar flow hood. Each plate was toothpick inoculated from an overnight colony and scored for motility after 18 hours incubation at 37°C [51]. Plates were visualized with a BioRad Geldoc system and digitally captured using BioRad Quantity One software.

### EPS precipitation

Cells were grown for 24 hours in TY Broth (LB broth supplemented after autoclaving with 10 mM MgSO_4_ and 100 µM MnSO_4_), pelleted, and the supernatant was put on ice. The chilled supernatant was mixed with ice cold 75% ethanol. For imaging in 12-well microtiter plates, the precipitate was mixed with glycerol to a final concentration of 17% and images were taken with a Canon Powershot A620 digital camera. For staining samples using Stains-All, the precipitate was spun down at 14,000 RPM for 3 minutes. The supernatant was discarded and the residual ethanol was allowed to evaporate. Each sample was mixed with 100µl of 1× SDS Sample Buffer and 10 µl was loaded onto a SDS-12% polyacrylamide gel. Samples were run for 30 minutes at 200 V. The stacking and resolving gel was fixed for 24 hours (25% isopropanol, 3% acetic acid) and stained overnight with 100 ml of Stains-All Reactive Solution (5 ml of 1 mg/ml Stains-All [Sigma] in formamide, 50 µl 2-mercaptoethanol, and 95 ml Stains All Base Solution [16.6% isopropanol, 5.5% formamide, and 0.5% 3.0M Tris-HCl (pH 8.8)]) [52]. The stacking gel was scanned using an HP Scanjet 4370.

For treatment with Proteinase K, 300 µl of supernatant was mixed with a final concentration of 400 µg/ml of Proteinase K (Fisher) for 1 hr at 55°C. For treatment with DNase and RNase, 300 µl of supernatant was mixed with a final concentration of 67 µg/ml DNase I (Roche) and 330 µg/ml Ribonuclease A (Sigma) for 30 minutes at 37°C. Both treated supernatants were then treated as described above to precipitate EPS.

### Generation of *epsE* mutant pool

To generate a pool of *epsE* mutants, primer pair 1386/1260 was used to amplify the *epsE* reading frame using 3610 chromosomal DNA as a template and Expand polymerase with Expand Buffer 1 (Roche). This fragment was digested with *NheI* and *BamHI* and ligated into the *NheI* and *BamHI* sites of pDP232 containing the P*_eps_* promoter region and a chloramphenical resistance cassette between the two arms of the *amyE* gene. Multiple ligations were transformed into *E. coli* and all of the resulting colonies were pooled to generate a plasmid library of *epsE^mut^* complementation constructs (pSG4). The plasmid library was transformed into naturally competent *B. subtilis* PY79 and phage lysates were generated. The phage lysates were used to transduce strains of *B. subtilis* for screening of enzymatic and clutch mutants of *epsE*.

### Isolation of Loss of Extracellular Polysaccharide mutants (*Lox*)

The *epsE^mut^* pool of lysates were transduced into a *sinR epsE* mutant (DS2174). The resulting smooth colonies were inoculated onto 0.7% LB agar plates and incubated at 37°C. Smooth colonies that were also inhibited for motility were isolated.

### Isolation of Loss of Clutch Function mutants (*Loc*)

The *epsE^mut^*pool of lysates were transduced into a *sinR eps* mutant (DS1722). All of the resulting colonies were pooled, spotted onto the center of 0.7% LB agar plates, and incubated at 37°C for approximately 6 hours. Motile cells were pooled from the edge of the swarm radius and phage lysates were generated. These phage lysates were transduced into a *sinR epsE* double mutant (DS2174). The rough colonies were isolated and a phage lysate was generated from each colony. These lysates were transduced into a *sinR ΔepsE epsH::tet* mutant (DS2946) to verify that each mutant *epsE^mut^* allele abolished clutch function.

### Sequencing *epsE*

The *epsE* mutants were sequenced by amplifying DNA from the appropriate strain using primer pair 953/345. The PCR product generated was sequenced using primers 732 and 733 individually.

### Anti-EpsE antibody preparation

1 µg of purified EpsE protein was submitted to Cocalico Biologicals for serial injection into a rabbit host for antibody generation. Anti-EpsE serum was mixed with EpsE-conjugated Affigel-10 beads and incubated overnight at 4°C. Beads were packed onto a 1 cm column (Bio-Rad) and then washed with 100 mM glycine (pH 2.5) to release the antibody and immediately neutralized with 2M unbuffered Tris. Affinity purification of the antibody was verified by SDS-PAGE. Purified antibody was dialyzed into 1× PBS, 50% glycerol and stored at -80°C.

### Western blotting

*B. subtilis* strains were grown in LB to OD_600_ ∼0.8, 10 ml were harvested by centrifugation, resuspended to 10 OD_600_ in Lysis buffer (20 mM Tris pH 7.0, 10 mM EDTA, 1 mg/ml lysozyme, 10 µg/ml DNAse I, 100 µg/ml RNAse I, 1 mM PMSF), and incubated for 30 minutes at 37°C. 10 µl of lysate was mixed with 2 µl 6× SDS loading dye. Samples were separated by 12% Sodium dodecyl sulfate-polyacrylamide gel electrophoresis (SDS-PAGE). The proteins were electroblotted onto nitrocellulose and developed with a 1∶10,000 dilution of primary antibody for anti-EpsE and a 1∶10,000 dilution of secondary antibody (horseradish peroxidase-conjugated goat anti-rabbit immunoglobulin G). Immunoblot was developed using the Immun-Star HRP developer kit (Bio-Rad). For blots probed with anti-SigA antibody, the samples used for the anti-EpsE western blot were diluted 1∶100 and a 1∶20,000 dilution of anti-SigA was used. Anti-SigA was a gift from Masaya Fujita (University of Houston).

### Microscopy

Fluorescence microscopy was performed with a Nikon 80i microscope with a phase contrast objective Nikon Plan Apo 100× and an Excite 120 metal halide lamp. FM4-64 was visualized with a C-FL HYQ Texas Red Filter Cube (excitation filter 532–587 nm, barrier filter >590 nm). GFP and Alexa Fluor 488 C_5_ maleimide fluorescent signals were visualized using a C-FL HYQ FITC Filter Cube (FITC, excitation filter 460–500 nm, barrier filter 515–550 nm). Images were captured with a Photometrics Coolsnap HQ2 camera in black and white, false colored, and superimposed using Metamorph image software.

For GFP microscopy, cells were grown overnight at 22°C in LB broth and 0.5 ml was pelleted and resuspended in 0.1 ml PBS. Membranes were stained by resuspension in 50 µl of PBS containing 5 µg/ml FM4-64 and incubated for 10 min at room temperature. Samples were observed by spotting 3 µl of the suspension on a glass microscope slide and were immobilized with a poly-L-lysine-treated glass coverslip.

For fluorescent microscopy of flagella, 0.5 ml of broth culture was harvested at 0.5–2.0 OD_600_, and washed once in 1.0 ml of T-Base Buffer (15 mM (NH_4_)_2_SO_4_, 80 mM K_2_HPO_4_, 44 mM KH_2_PO_4_, 3.4 mM sodium citrate, and 3.0 mM MgSO_4_·6H_2_0). The suspension was pelleted, resuspended in 50 µl of T-Base buffer containing 5µg/ml Alexa Fluor 488 C_5_ maleimide (Molecular Probes), and incubated for 5 min at room temperature [17]. Cells were then washed twice with 500 µl T-Base buffer. Membranes were stained by resuspension in 50 µl of T-Base buffer containing 5 µg/ml FM4-64 (Molecular Probes) and incubated for 5 min at room temperature. Three microliters of suspension were placed on a microscope slide and immobilized with a poly-L-lysine-treated coverslip.

### Isolation of suppressors that regain pellicle biomass

Three replicates of strains DS1674 and DS4532 were inoculated into MSgg, grown at 25°C, and images were taken every 24 hours for 4 days. The pellicles were harvested and dilution plated for discrete colony forming units. Three hundred colonies of each replicate were inoculated onto 0.7% swarm agar and incubated at 37°C. After 5 hours, the number of colonies that were non-motile were counted.

### EpsE predicted structure

The amino acid sequence of EpsE from *B. subtilis* strain 3610 was submitted to 3D-JIGSAW (version 2.0) [53]–[55].

### Strain construction

All constructs were first introduced into the domesticated strain PY79 by natural competence and then transferred to the 3610 background using SPP1-mediated generalized phage transduction [56]. All strains used in this study are listed in Table S2. All plasmids used in this study are listed in Table S3. All primers used in this study are listed in Table S4.

#### Complementation constructs

To generate the *amyE::P_eps_-epsE^D94A^* complementation construct (pKB30), *epsE^D94A^* was amplified from DS2606 with primer pair 712/713 and digested with *NheI* and *BamHI*. The digested *epsE^D94A^* PCR fragment was ligated into the *NheI/BamHI* sites of pDP232 containing the P*eps* promoter and a chloramphenical resistance gene between the arms of the *amyE* gene.

#### Translational fusions

To generate the translational fusion of EpsE to GFP, a fragment containing *P_eps_-epsE* was amplified using DS4734 (*epsE^Y197C^*), DS4777 (*epsE^K106E^*), DS4741 (*epsE^F110L^*), and DS4782 (*epsE^K113E^*) as templates and primer pair 709/975 and was digested with *EcoRI* and *XhoI*. The *gfp* gene was amplified from pKB49 with primer pair 995/996 and was digested with *XhoI* and *BamHI*. Both digested fragments were simultaneously ligated into the *EcoRI* and *BamHI* sites of pDG1664 [57] containing a erythromycin (*mls*) resistance cassette and a polylinker downstream between the arms of the *thrC* gene to generate plasmids pSG9, pSG11, pSG17, and pSG19. A flexible linker domain was incorporated between EpsE and GFP in the fusion protein [58].

#### SPP1 phage transduction

To 0.2 ml of dense culture grown in TY broth (LB broth supplemented after autoclaving with 10 mM MgSO_4_ and 100 µM MnSO_4_), serial dilutions of SPP1 phage stock were added and statically incubated for 15 minutes at 37°C. To each mixture, 3 ml TYSA (molten TY supplemented with 0.5% agar) was added, poured atop fresh TY plates, and incubated at 30°C overnight. Top agar from the plate containing near confluent plaques was harvested by scraping into a 15 ml conical tube, treated with 25 µg/ml DNase final concentration, vortexed and centrifuged at 5,000×g for 10 minutes. The supernatant was passed through a 0.45 µm syringe filter and stored at 4°C.

Recipient cells were grown to stationary phase in 3 ml TY broth at 37°C. 1.0 ml cells were mixed with 25 µl of SPP1 donor phage stock. 9 ml of TY broth was added to the mixture and incubated statically at 37°C for 30 minutes. The transduction mixture was then centrifuged at 5,000×g for 10 minutes, the supernatant was discarded and the pellet was resuspended in the remaining volume. 100 µl of cell suspension was then plated on TY fortified with 1.5% agar, the appropriate antibiotic, and 10 mM sodium citrate.

## Supporting Information

Figure S1 EpsE and FliG conservation. The black shaded boxes indicate identical conservation of residues between sequences while grey shaded boxes indicate conservation of similar residues between sequences. A) The primary sequence of *B. subtilis* EpsE is compared to other glycosyltransferases. The sites of *lox* mutations are indicated with red circles. The sites of *loc* mutations are indicated by green circles. The bacterial species are as follows: Bsu - *B. subtilis*, Lde - *Lactobacillus delbrueckii*, Bce - *Bacillus cereus*, Eco - *Escherichia coli*, Vch - *Vibrio cholerae*, Sme - *Sinorhizobium meliloti*. B) The primary sequence of *B. subtilis* FliG is compared to rotor proteins of other organisms. Clutch-insusceptible mutations are indicated with purple circles. The bacterial species are as follows: Bsu - *B. subtilis*, Tma - *Thermotoga maritima*, Eco - *E. coli*, Lmo - *Listeria monocytogenes*, Pae - *Pseudomonas aeruginosa*, Rsp - *Rhodobacter sphaeroides*, Vch - *V. cholerae*.
(3.03 MB TIF)

Figure S2 EpsE *loc* mutants. Alignment of all the isolates that lost clutch function and retained EPS biosynthesis with the EpsE^WT^ sequence. A) EpsE protein sequences B) EpsE DNA sequences
(3.31 MB TIF)

Figure S3 The *fliG^V338A^* clutch-insusceptible allele does not change the amount of EpsE produced in the cell. Whole cell lysates of cells mutated for *sinR epsH* (DS1674) and the indicated *fliG^V338A^* clutch-insusceptible allele (DS4532) were separately probed with anti-EpsE antibody and anti-SigA antibody in Western blot analysis (to serve as a loading control).
(0.19 MB TIF)

Figure S4 EPS and clutch function synergize in the *sinR* mutant but not wild type to promote pellicle formation. Images depict top-down views of 6-well microtiter plates containing MSgg media and the indicated strains incubated for 2 days at 25°C. Scale bar equals 1 cm. A. The strains are as follow: wild type (3610), *ΔepsE* (DS2152), *epsH fliG^V338A^* (DS3298), and *epsH* (DS76). B. *sinR epsE^D94A^ fliG^V338A^* (DS3394) and media alone.
(1.56 MB TIF)

Table S1 *lox* alleles.
(0.04 MB DOC)

Table S2 Strains.
(0.11 MB DOC)

Table S3 Plasmids.
(0.03 MB DOC)

Table S4 Primers.
(0.03 MB DOC)
