# Supplementary figures and images for: The EpsE Flagellar Clutch Is Bifunctional and Synergizes with EPS Biosynthesis to Promote Bacillus subtilis Biofilm Formation
Source: PLoS Genet. 2010 Dec 9;6(12):e1001243. doi: 10.1371/journal.pgen.1001243 (PMC3000366; doi:10.1371/journal.pgen.1001243)

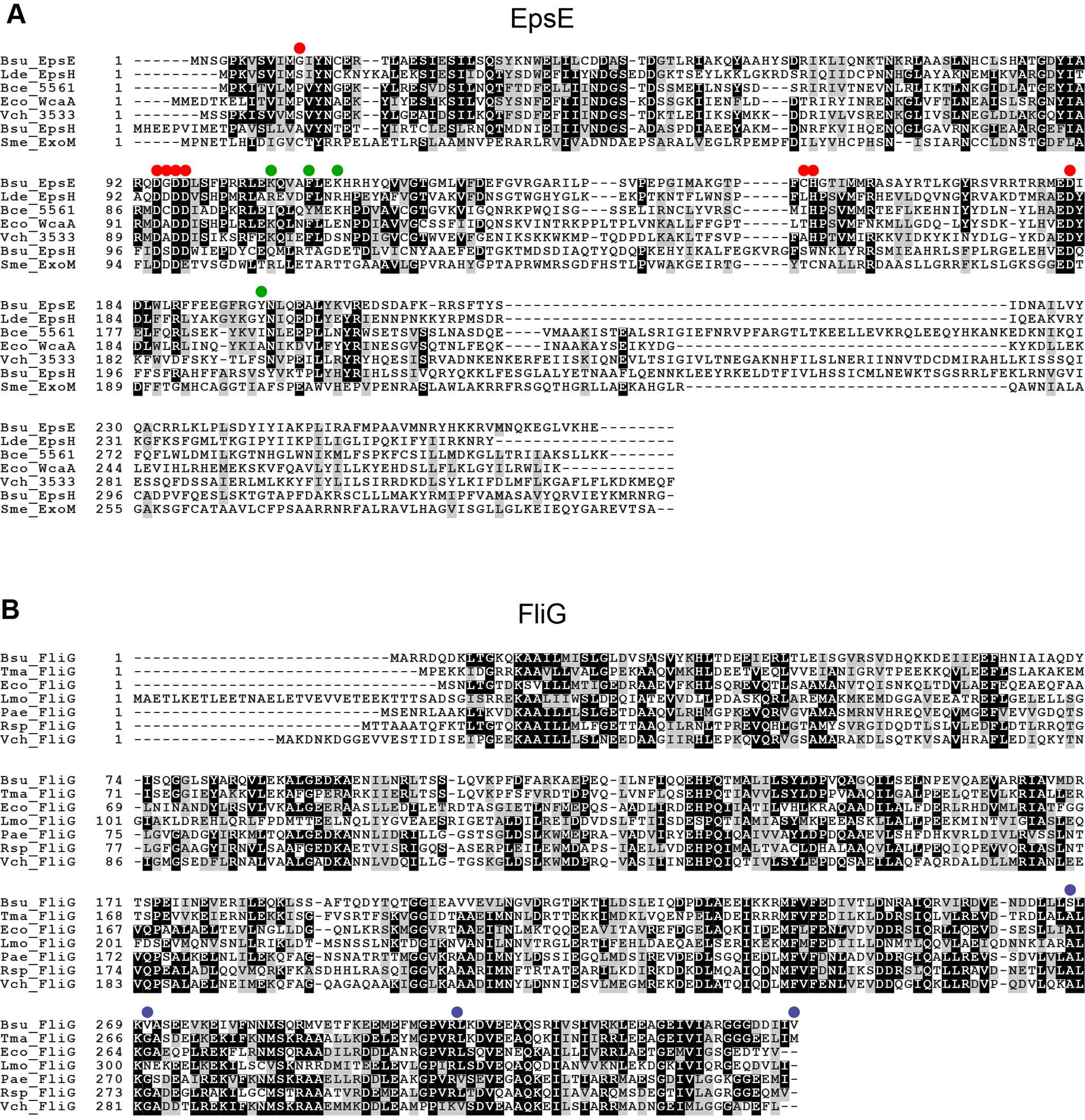

Supplement: Figure S1 — EpsE and FliG conservation. The black shaded boxes indicate identical conservation of residues between sequences while grey shaded boxes indicate conservation of similar residues between sequences. A) The primary sequence of B. subtilis EpsE is compared to other glycosyltransferases. The sites of lox mutations are indicated with red circles. The sites of loc mutations are indicated by green circles. The bacterial species are as follows: Bsu - B. subtilis, Lde - Lactobacillus delbrueckii, Bce - Bacillus cereus, Eco - Escherichia coli, Vch - Vibrio cholerae, Sme - Sinorhizobium meliloti. B) The primary sequence of B. subtilis FliG is compared to rotor proteins of other organisms. Clutch-insusceptible mutations are indicated with purple circles. The bacterial species are as follows: Bsu - B. subtilis, Tma - Thermotoga maritima, Eco - E. coli, Lmo - Listeria monocytogenes, Pae - Pseudomonas aeruginosa, Rsp - Rhodobacter sphaeroides, Vch - V. cholerae. (3.03 MB TIF) [file pgen.1001243.s001.tif]

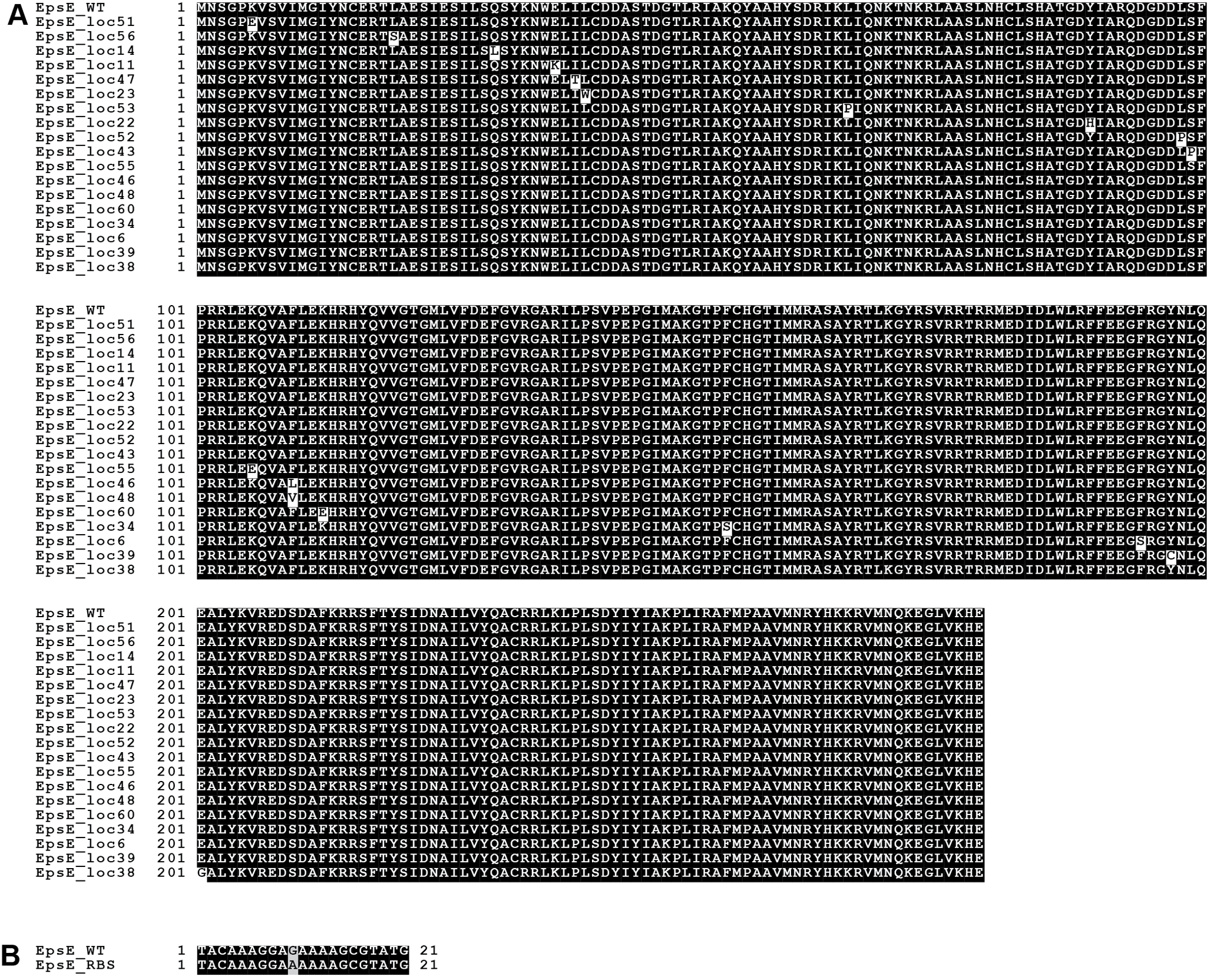

Supplement: Figure S2 — EpsE loc mutants. Alignment of all the isolates that lost clutch function and retained EPS biosynthesis with the EpsEWT sequence. A) EpsE protein sequences B) EpsE DNA sequences (3.31 MB TIF) [file pgen.1001243.s002.tif]

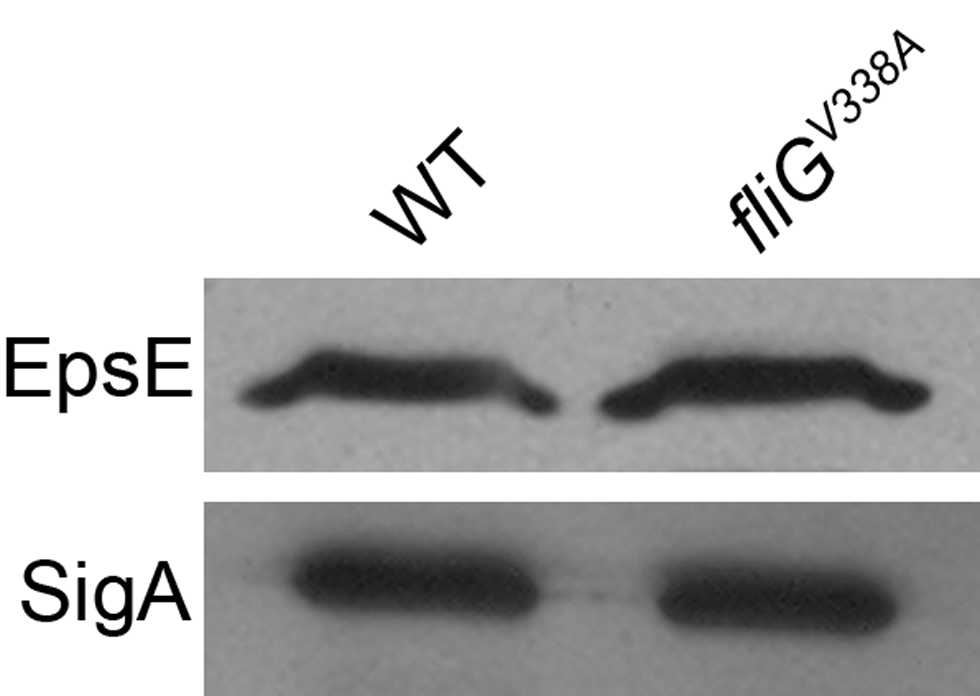

Supplement: Figure S3 — The fliGV338A clutch-insusceptible allele does not change the amount of EpsE produced in the cell. Whole cell lysates of cells mutated for sinR epsH (DS1674) and the indicated fliGV338A clutch-insusceptible allele (DS4532) were separately probed with anti-EpsE antibody and anti-SigA antibody in Western blot analysis (to serve as a loading control). (0.19 MB TIF) [file pgen.1001243.s003.tif]

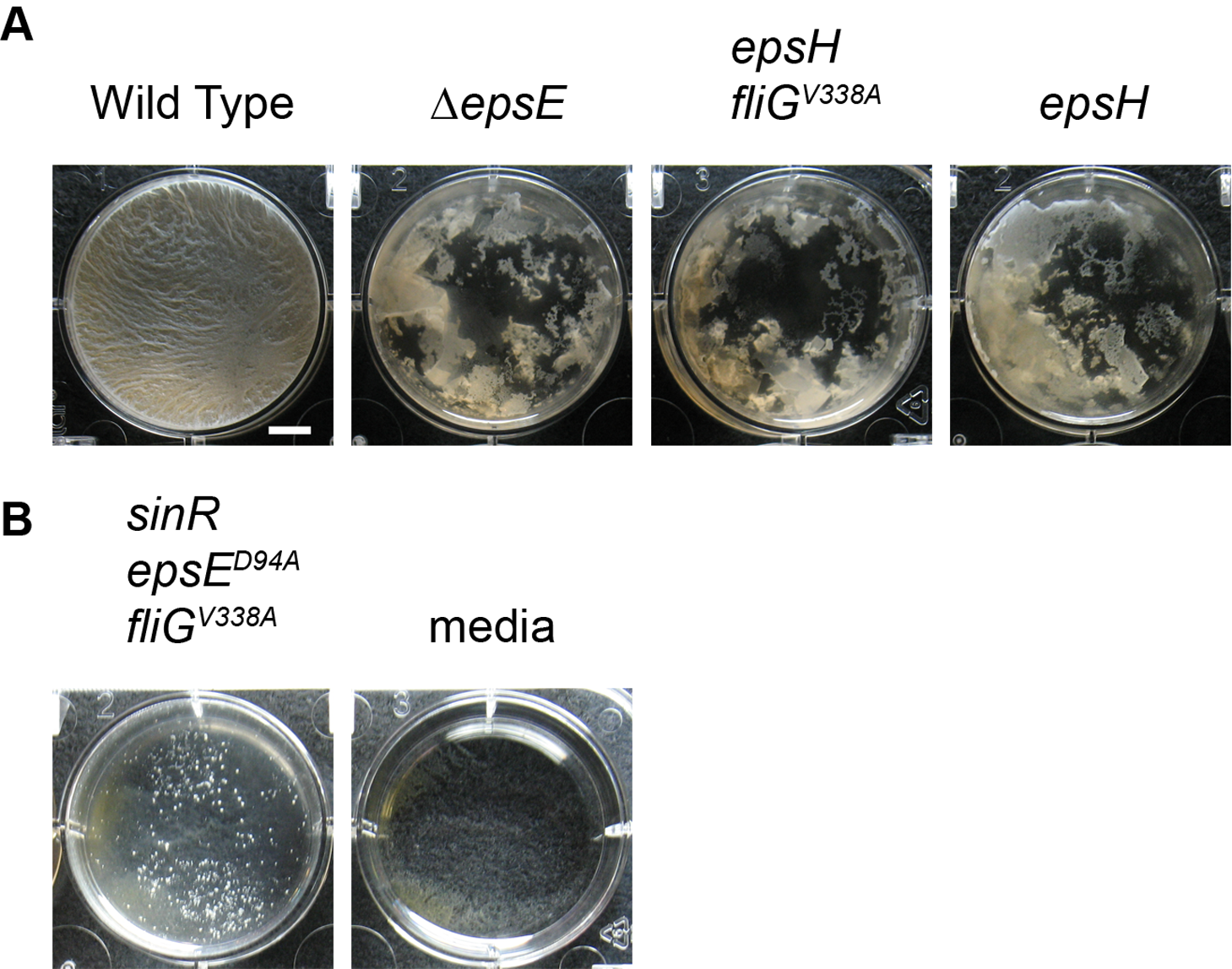

Supplement: Figure S4 — EPS and clutch function synergize in the sinR mutant but not wild type to promote pellicle formation. Images depict top-down views of 6-well microtiter plates containing MSgg media and the indicated strains incubated for 2 days at 25°C. Scale bar equals 1 cm. A. The strains are as follow: wild type (3610), ΔepsE (DS2152), epsH fliGV338A (DS3298), and epsH (DS76). B. sinR epsED94A fliGV338A (DS3394) and media alone. (1.56 MB TIF) [file pgen.1001243.s004.tif]
