# Supplementary material for: The EpsE Flagellar Clutch Is Bifunctional and Synergizes with EPS Biosynthesis to Promote Bacillus subtilis Biofilm Formation
Source: PLoS Genet. 2010 Dec 9;6(12):e1001243. doi: 10.1371/journal.pgen.1001243 (PMC3000366; doi:10.1371/journal.pgen.1001243)
Supplement: Table S1 — lox alleles. (0.04 MB DOC) [file pgen.1001243.s005.doc]

**Table S1: *lox* alleles**

| Strain | Allele | Mutation |
| --- | --- | --- |
| Class I: The DXDD active site motif | | |
| DS2287 | *lox1* | D94A |
| DS5841 | *lox9* | D97G |
| Class II: Other residues required for activity | | |
| DS5848 | *lox16* | G12D |
| DS5839 | *lox7* | C154R |
| DS5852 | *lox20* | H155R |
| DS5837 | *lox5* | D182G |
| Class III: Multiple mutations^a^ | | |
| DS5849 | *lox17* | S77P, **D94G** |
| DS5862 | *lox30* | **G95R**, A257T |
| DS5855 | *lox23* | **D96G**, I222S |
| DS5860 | *lox28* | **D96G**, N224D |
| DS5856 | *lox24* | **D97G**, M159I |
| DS5858 | *lox26* | **C154R**, F190L |
| DS5840 | *lox8* | **C154W**, H263Y |
| DS5838 | *lox6* | Q54R, **H155R** |
| DS5844 | *lox12* | T167A, **D182N** |

^a^Bolded mutation is a mutation that corresponds to either a

Class I or Class II mutation and is predicted to be responsible

for the *lox* phenotype.
