# Supplementary material for: The EpsE Flagellar Clutch Is Bifunctional and Synergizes with EPS Biosynthesis to Promote Bacillus subtilis Biofilm Formation
Source: PLoS Genet. 2010 Dec 9;6(12):e1001243. doi: 10.1371/journal.pgen.1001243 (PMC3000366; doi:10.1371/journal.pgen.1001243)
Supplement: Table S2 — Strains. (0.11 MB DOC) [file pgen.1001243.s006.doc]

**Table S2: Strains**

| Strain | Genotype Reference |
| --- | --- |
| PY79 | Lab strain |
| 3610 | Wild type |
| DS76 | *epsH::tet (10)* |
| DS859 | *sinR::kan (17)* |
| DS991 | *sinR::kan tasA::Tn10 spec* |
| DS1674 | *sinR::kan epsH::tet (17)* |
| DS1722 | *sinR::kan epsA-O::tet* |
| DS2152 | *∆epsE* |
| DS2174 | *sinR::kan ∆epsE* |
| DS2179 | *sinR::kan epsH::tet amyE::P_hag_-hag^T209C^ spec* |
| DS2239 | *sinR::kan ∆epsE amyE::P_eps_-epsE cat* |
| DS2287 | *sinR::kan ∆epsE amyE::P_eps_-epsE^D94A^ cat* |
| DS2369 | *sinR::kan tasA::Tn10 spec ∆epsE* |
| DS2370 | *sinR::kan tasA::Tn10 spec ∆epsE amyE::P_eps_-epsE cat* |
| DS2372 | *sinR::kan tasA::Tn10 spec ∆epsE amyE::P_eps_-epsE^D94A^ cat* |
| DS2606 | *amyE::P_hyspank_-epsE^D94A^ spec* |
| DS2946 | *sinR::kan epsH::tet ∆epsE (17)* |
| DS2989 | *sinR::kan ∆epsE thrC::P_eps_-epsE-GFP mls (17)* |
| DS3004 | *sinR::kan ∆epsE fliG^V338A^ thrC::P_eps_-epsE-GFP mls (17)* |
| DS3005 | *sinR::kan fliG^V338A^* |
| DS3298 | *epsH:tet fliG^V338A^* |
| DS3394 | *sinR::kan fliG^V338A^ ∆epsE amyE::P_eps_-epsE^D94A^ cat* |
| DS3844 | *sinR::kan ∆epsE epsH::tet amyE::P_eps_-epsE^WT^ cat (17)* |
| DS4275 | *sinR::kan ∆epsE amyE::P_eps_-epsE^RBS^ (loc2) cat* |
| DS4284 | *sinR::kan ∆epsE amyE::P_eps_-epsE^F194S^ (loc 6) cat* |
| DS4289 | *sinR::kan ∆epsE amyE::P_eps_-epsE^E36K^ (loc 11) cat* |
| DS4532 | *sinR::kan fliG^V338A^ epsH::tet* |
| DS4593 | *sinR::kan ∆epsE epsH::tet amyE::P_eps_-epsE^D94A^ cat* |
| DS4689 | *sinR::kan ∆epsE amyE::P_eps_-epsE^Q30L^ (loc 14) cat* |
| DS4697 | *sinR::kan ∆epsE amyE::P_eps_-epsE^Y89H^ (loc 22) cat* |
| DS4698 | *sinR::kan ∆epsE amyE::P_eps_-epsE^L39W^ (loc 23) cat* |
| DS4729 | *sinR::kan ∆epsE amyE::P_eps_-epsE^F153S^ (loc 34) cat* |
| DS4733 | *sinR::kan ∆epsE amyE::P_eps_-epsE^E201G^ (loc 38) cat* |
| DS4734 | *sinR::kan ∆epsE amyE::P_eps_-epsE^Y197C^ (loc 39) cat* |
| DS4738 | *sinR::kan ∆epsE amyE::P_eps_-epsE^S99P^ (loc 43) cat* |
| DS4741 | *sinR::kan ∆epsE amyE::P_eps_-epsE^F110L^ (loc 46) cat* |
| DS4742 | *sinR::kan ∆epsE amyE::P_eps_-epsE^I38T^ (loc 47) cat* |
| DS4743 | *sinR::kan ∆epsE amyE::P_eps_-epsE^F110V^ (loc 48) cat* |
| DS4746 | *sinR::kan ∆epsE amyE::P_eps_-epsE^K6E^ (loc 51) cat* |
| DS4747 | *sinR::kan ∆epsE amyE::P_eps_-epsE^L98P^ (loc 52) cat* |
| DS4748 | *sinR::kan ∆epsE amyE::P_eps_-epsE^L65P^ (loc 53) cat* |
| DS4777 | *sinR::kan ∆epsE amyE::P_eps_-epsE^K106E^ (loc 55) cat* |
| DS4778 | *sinR::kan ∆epsE amyE::P_eps_-epsE^L20S^ (loc 56) cat* |
| DS4782 | *sinR::kan ∆epsE amyE::P_eps_-epsE^K113E^ (loc 60) cat* |
| DS5086 | *sinR::kan ∆epsE epsH::tet amyE::P_eps_-epsE^K113E^ (loc 60) cat* |
| DS5087 | *sinR::kan ∆epsE epsH::tet amyE::P_eps_-epsE^Y197C^ (loc 39) cat* |
| DS5088 | *sinR::kan ∆epsE epsH::tet amyE::P_eps_-epsE^I38T^ (loc 47) cat* |
| DS5089 | *sinR::kan ∆epsE epsH::tet amyE::P_eps_-epsE^RBS^ (loc 2) cat* |
| DS5090 | *sinR::kan ∆epsE epsH::tet amyE::P_eps_-epsE^S99P^ (loc 43) cat* |
| DS5135 | *sinR::kan ∆epsE epsH::tet amyE::P_eps_-epsE^F194S^ (loc 6) cat* |
| DS5136 | *sinR::kan ∆epsE epsH::tet amyE::P_eps_-epsE^F110V^ (loc 48) cat* |
| DS5137 | *sinR::kan ∆epsE epsH::tet amyE::P_eps_-epsE^E36K^ (loc 11) cat* |
| DS5138 | *sinR::kan ∆epsE epsH::tet amyE::P_eps_-epsE^L39W^ (loc 23) cat* |
| DS5139 | *sinR::kan ∆epsE epsH::tet amyE::P_eps_-epsE^Q30L^ (loc 14) cat* |
| DS5140 | *sinR::kan ∆epsE epsH::tet amyE::P_eps_-epsE^L20S^ (loc 56) cat* |
| DS5141 | *sinR::kan ∆epsE epsH::tet amyE::P_eps_-epsE^Y89H^ (loc 22) cat* |
| DS5142 | *sinR::kan ∆epsE epsH::tet amyE::P_eps_-epsE^K106E^ (loc 55) cat* |
| DS5143 | *sinR::kan ∆epsE epsH::tet amyE::P_eps_-epsE^F110L^ (loc 46) cat* |
| DS5144 | *sinR::kan ∆epsE epsH::tet amyE::P_eps_-epsE^L98P^ (loc 52) cat* |
| DS5145 | *sinR::kan ∆epsE epsH::tet amyE::P_eps_-epsE^E201G^ (loc 38) cat* |
| DS5153 | *sinR::kan ∆epsE epsH::tet amyE::P_eps_-epsE^F153S^ (loc 34) cat* |
| DS5154 | *sinR::kan ∆epsE epsH::tet amyE::P_eps_-epsE^K6E^ (loc 51) cat* |
| DS5155 | *sinR::kan ∆epsE thrC::P_eps_-epsE^Y197C^ (loc 39)-GFP mls* |
| DS5156 | *sinR::kan ∆epsE thrC::P_eps_-epsE^K106E^ (loc 55)-GFP mls* |
| DS5185 | *sinR::kan ∆epsE epsH::tet amyE::P_eps_-epsE^L65P^ (loc 53) cat* |
| DS5187 | *sinR::kan tasA::Tn10 spec epsH::tet* |
| DS5489 | *sinR::kan ∆epsE thrC::P_eps_-epsE^F110L^ (loc 46)-GFP mls* |
| DS5491 | *sinR::kan ∆epsE thrC::P_eps_-epsE^K113E^ (loc 60)-GFP mls* |
| DS5837 | *sinR::kan ∆epsE amyE::P_eps_-epsE^D182G^ (lox 5) cat* |
| DS5839 | *sinR::kan ∆epsE amyE::P_eps_-epsE^C154R^ (lox 7) cat* |
| DS5841 | *sinR::kan ∆epsE amyE::P_eps_-epsE^D97G^ (lox 9) cat* |
| DS5848 | *sinR::kan ∆epsE amyE::P_eps_-epsE^G12D^ (lox 16) cat* |
| DS5852 | *sinR::kan ∆epsE amyE::P_eps_-epsE^H155R^ (lox 20) cat* |
| DS6190 | *sinR::kan tasA::Tn10 spec ∆epsE amyE::P_eps_-epsE^D182G^ (lox 5) cat* |
| DS6191 | *sinR::kan tasA::Tn10 spec ∆epsE amyE::P_eps_-epsE^G12D^ (lox 16) cat* |
| DS6195 | *sinR::kan tasA::Tn10 spec ∆epsE amyE::P_eps_-epsE^D97G^ (lox 9) cat* |
| DS6312 | *sinR::kan tasA::Tn10 spec ∆epsE amyE::P_eps_-epsE^K106E^ (loc 55)cat* |
| DS7147 | *sinR::kan tasA::Tn10 spec ∆epsE amyE::P_eps_-epsE^RBS^ (loc 2) cat* |
| DS7289 | *sinR::kan epsH::tet fliG^V338A^ amyE::P_hag_-hag^T209C^ spec* |
| DS7290 | *sinR::kan epsH::tet 4 day isolate amyE::P_hag_-hag^T209C^ spec* |
| DS7293 | *sinR::kan epsH::tet fliG^V338A^ 4 day non-motile isolate amyE::P_hag_-hag^T209C^ spec* |
