# Supplementary material for: The EpsE Flagellar Clutch Is Bifunctional and Synergizes with EPS Biosynthesis to Promote Bacillus subtilis Biofilm Formation
Source: PLoS Genet. 2010 Dec 9;6(12):e1001243. doi: 10.1371/journal.pgen.1001243 (PMC3000366; doi:10.1371/journal.pgen.1001243)
Supplement: Table S3 — Plasmids. (0.03 MB DOC) [file pgen.1001243.s007.doc]

Table S3: Plasmids

| Plasmid | Genotype Reference |
| --- | --- |
| pDG1664 | *thrC::mls amp (57)* |
| pDP232 | *amyE::P_eps_-epsE cat amp (17)* |
| pKB30 | *amyE::P_eps_-epsE^D94A^ cat amp* |
| pKB59 | *thrC::P_eps_epsE^WT^-GFP mls amp (17)* |
| pSG4 | *amyE::P_eps_-epsE^mut^ cat amp* |
| pSG9 | *thrC::P_eps_-epsE^Y197C^ (loc 39)-GFP mls amp* |
| pSG11 | *thrC::P_eps_-epsE^K106E^ (loc 55)-GFP mls amp* |
| pSG17 | *thrC::P_eps_epsE^F110L^(loc 46)-GFP mls amp* |
| pSG19 | *thrC::P_eps_-epsE^K113E^(loc 60)-GFP mls amp* |
