# Supplementary material for: The EpsE Flagellar Clutch Is Bifunctional and Synergizes with EPS Biosynthesis to Promote Bacillus subtilis Biofilm Formation
Source: PLoS Genet. 2010 Dec 9;6(12):e1001243. doi: 10.1371/journal.pgen.1001243 (PMC3000366; doi:10.1371/journal.pgen.1001243)
Supplement: Table S4 — Primers. (0.03 MB DOC) [file pgen.1001243.s008.doc]

Table S4: Primers

| Primer | Sequence |
| --- | --- |
| 345 | GTCGTTATTTCGTTCATTATAAGGAATT |
| 709 | AGGAGGAATTCTGTACGGCTTGCACTAAATGTACG |
| 712 | AGGAGGCTAGCAAAGGAGAAAAGCGTATGAACTCAG |
| 713 | CTCCTGGATCCTGGCTGCTATTCATGCTTGACAAG |
| 732 | GCGCCGTCTGGAAAAGCAGGTC |
| 733 | GCCCGGCTCCGGAACAGAAGG |
| 953 | GGAGTGTCAAGAATGTTTGCAAAAC |
| 975 | CTCCTCTCGAGGCCAGAACCAGCAGCGGAGCCAGCGGATCCTTCATGCTTGACAAGCCCTTCCTTTT |
| 995 | AGGAGCTCGAGGGTTCCGGAATGAGTAA |
| 996 | CTCCTGGATCCTTATTTGTATAGTTCATCCATGCCA |
| 1260 | CTCCTGGATCCCTGAGAACATGGAGCACGCGC |
| 1386 | AGGAGGCTAGCAATGGCAGATATTTATTCCGCGTAC |
